# Introduction of quality management in a National Reference Laboratory in Germany

**DOI:** 10.1371/journal.pone.0222925

**Published:** 2019-10-15

**Authors:** Susanne Homolka, Julia Zallet, Heidi Albert, Anne-Kathrin Witt, Katharina Kranzer

**Affiliations:** 1 National Reference Laboratory for Mycobacteria, Research Center Borstel, Borstel, Germany; 2 Molecular and Experimental Mycobacteriology, Research Center Borstel, Borstel, Germany; 3 Foundation for Innovative New Diagnostics (FIND) South Africa, Cape Town, South Africa; 4 Clinical Research Department, London School of Hygiene and Tropical Medicine, London, England, United Kingdom; School of Pathology, National Health Laboratory Service (NHLS) and University of the Witwatersrand, South Africa, SOUTH AFRICA

## Abstract

**Background:**

High quality diagnostic services are crucial for tuberculosis (TB) diagnosis, treatment and control. A strong laboratory quality management system (QMS) is critical to ensuring the quality of testing and results. Recent initiatives to improve TB laboratory quality have focused on low and middle-income countries, but similar issues also apply to high-income countries.

**Methods and findings:**

Using a multipronged approach reviews of facilities, equipment, processes (purchasing, pre-analytic, analytic and post-analytic), staff, health and safety, documentation, information management and organization based on the ISO 15189 and the twelve quality system essentials were conducted between October 2015 and January 2016 at the National TB Reference Laboratory in Germany. Outcome assessment included proportion of smear positive slides, proportion of contaminated liquid cultures and DNA contamination rates before and after implementation of QMS. The odds ratio for these outcomes was calculated using a before/after comparison. Reviews highlighted deficiencies across all twelve quality system essentials and were addressed in order of priority and urgency. Actions aimed at improving analytical quality, health and safety and information management were prioritised for initial implementation in parallel with each other. The odds ratio for a sample to be tested as microscopically positive increased by 2.08 (95%CI 1.41–3.06) comparing the time before with the time after implementation of quality managed fluorescence microscopy. Liquid culture contamination rates decreased from 23.6- 7.6% in April-July 2016 to <10% in November 2017-March 2018. The proportion of negative controls showing evidence of DNA contamination decreased from 38.2% in 2013 to 8.1% in 2017, the corresponding odds ratio was 0.14 (95%CI 0.07–0.29).

**Conclusion:**

This study showed marked improvement on quality indicators after implementation of a QMS in a National TB Reference Laboratory. The challenges and lessons learned in this study are valuable not just for high-income settings, but are equally generalizable to other laboratories.

## Introduction

High-quality laboratory services are an essential component for tuberculosis care and control.[1] New diagnostic methods and increasing awareness about medical errors and their consequences emphasizes the great importance of quality in health.[2] A strong laboratory quality management system (QMS) is critical to ensuring the quality of testing.

Over the last six decades laboratory quality management has experienced ongoing development.[3] A ten-fold reduction in the analytical error rate has been achieved over the past decade as a result of improved reliability and standardization of analytical techniques, reagents, and instrumentation especially in clinical chemistry.[4–6] In addition, advances in information technology, quality control and quality assurance methods have also led to error reduction.

Laboratory quality management is generally accepted as state of the art practise and is legally prescribed in many countries such as Germany and the USA.[7, 8] Integral parts of a QMS, often implemented in the early stages of an improvement initiative, include quality control (QC), external quality assessment (EQA), standard operating procedures (SOP) and competency assessment (CA). Several studies have shown that QMS implementation results in a measurable improvement in the quality of services and increased patient safety due to a reduction in laboratory errors.[4, 9–11]

Germany is a low tuberculosis incident country with a total of 5915 tuberculosis patients notified in 2016 amounting to an annual tuberculosis notification rate of 7.2 cases per 100 000 population.[12] Microbiological confirmation was obtained for 71.5% of all cases. The laboratory network in Germany comprises stand-alone microbiology laboratories run by district, town or university hospitals, private laboratories and private laboratory companies operating either regionally or nationally. Approximately 150 and 50 laboratories perform mycobacterial culture and tuberculosis drug susceptibility testing (DST) respectively. Germany has an insurance-based health care system with fixed prices for diagnostic tests. Different prices are applied for in- and outpatients and for patient insured by standard or private insurance. Laboratories are at liberty to offer discount packages. The National Mycobacterium Reference Laboratory (NRL) was appointed by the Ministry of Health through consultation with the Robert Koch Institute. The NRL receives some federal funding and generates revenue through diagnostic procedures.

The NRL has been implementing a comprehensive and data-driven approach to improving quality management systems at the laboratory, starting in 2015 with a detailed laboratory review process. This review led to adoption of a phased implementation approach starting with introduction of QM and health and safety policies, and building renovations. In 2017, a laboratory information system (LIS) with audit trails, automated quality statistics and barcodes was introduced. Here we present our experiences of implementing a QMS, challenges and lessons learned and the impact of the interventions based on measurement of trends in key quality indicators before, during and after implementation of various aspects of the QMS.

## Methods

### National Reference Laboratory

The NRL receives 12 000–15 000 samples every year. The samples are referred by the regional referral hospital specializing in respiratory diseases (including tuberculosis and specifically drug resistant tuberculosis) (30%), hospitals and private practitioners regionally (15%) and other laboratories nationally (40%). The remaining 15% of samples are sent to the NRL in its capacity as a WHO (World Health Organisation) supranational reference laboratory from partner countries internationally. These samples are referred for DST as part of quality assurance for national drug resistance surveys or for second line DST for rifampicin resistant isolates from countries without local DST capacity. The NRL in partnership with INSTAND (Gesellschaft zur Förderung der Qualitätssicherung in medizinischen Laboratorien e.V) runs the twice-yearly mycobacterial EQAs for Germany and through a European Center for Disease Prevention and Control (ECDC) funded scheme for countries in the European Union.

The NRL employs a director, two scientists, seven full-time and two part-time technicians, two part-time laboratory assistants and two administrators.

Diagnostic procedures at the NRL include smear microscopy, solid and liquid mycobacterial culture, direct molecular tests to detect non-tuberculosis mycobacterial (NTM) DNA, *Mycobacterium tuberculosis* complex DNA and resistance conferring mutations, species identification, genotypic and phenotypic DST. The laboratory infrastructure includes biosafety level II and III laboratories (BSL II and III).[13]

### Laboratory review

A series of laboratory reviews was conducted between October 2015 and January 2016, prompted by a change in laboratory leadership. Using a multipronged approach reviews of facilities, equipment, processes (purchasing, pre-analytic, analytic and post-analytic), staff, health and safety, documentation, information management and organization based on the ISO 15189 and the twelve quality system essentials were conducted.[14]

The newly appointed clinical laboratory director together with an external quality management consultant reviewed all available documents, directly observed sample processing and analytic procedures, interviewed staff working in the laboratory and at core facilities (human resources and central procurement). The results of the review were summarized in a narrative report and a list of priorities areas for actions was drawn up. This was presented to the institutional senior management resulting in additional resources for process optimization, infrastructure, staff and laboratory information and data management.

In addition, the laboratory manager (previous quality manager) of the English National Mycobacterial Reference Laboratory was invited for a 3-day visit to identify areas for improvement mainly focused on analytic processes and health and safety of employees. Findings were summarized in a supervisory report, presented both to senior management and laboratory staff. The institutional health and safety officer, together with the head of technical services and the clinical laboratory director undertook a detailed risk assessment aligned with the German biosafety regulations.[15] In addition, a ventilation engineer was asked to review the ventilation system and measure pressure differences and air exchanges.

### Actions

Following the reviews, a quality manager and a quality management technician were employed. One of the technicians was promoted to acting laboratory manager and funded to undertake a two-year part-time course in laboratory management. As a result of the risk assessment and the ventilation engineer report, a laboratory planner and architect was commissioned to assess the feasibility of refurbishing existing facilities in order to comply with the 2013-biosafety regulations.

Because the baseline reviews had identified deficiencies across all aspects of the twelve quality management essentials, the decision was taken to address deficiencies in order of priority and urgency. Actions aimed at improving analytical quality, health and safety and information management were prioritised for initial implementation in parallel with each other.

### Outcomes

Impact was measured using quality indicators across three analytic methods before and after implementing improvement activities: i) smear microscopy ii) liquid mycobacterial culture and iii) molecular DST. Patient population, referring hospitals and sample numbers were comparable across different time periods. The prevalence of positive cultures for mycobacteria (*M*. *tuberculosis* complex or non-tuberculous mycobacteria) was not significantly different before and after the intervention (p = 0.10).

The proportion of smear positive slides from respiratory samples and the proportion of false positive samples were calculated before, during and after implementing improvement activities. Differences in proportions were compared using χ^2^ test. Odds ratios for smear positivity were calculated using the pre-implementation period as the baseline. Only the first respiratory sample of a patient was included in the analysis. A false positive smear was defined as a smear categorized as positive in treatment naïve patients without detection of mycobacterial DNA in the primary sample, no mycobacterial growth in liquid media after 6 weeks and solid culture after 8 weeks.

As part of the baseline review the proportions of contaminated liquid cultures were determined retrospectively over three consecutive months (February-May) and years (2013–2015) by extracting data manually from paper records In line with routine laboratory procedures positive liquid cultures were investigated using the standard Ziehl-Neelsen stain and light microscopy, sub-culture on Columbia blood agar and Loewenstein-Jensen slants and molecular diagnostics aimed at detecting non-tuberculosis mycobacterial or *M*. *tuberculosis* complex DNA. Our limited data collection allowed investigation into possible monthly and yearly variation while limiting the staff time required for the data extraction. Routine prospective collection of contamination rates as quality indicators was implemented in April 2016. According to German microbiology standards contamination rates were calculated for sputum samples only and restricted to treatment naïve patients, not known to have cystic fibrosis. [16] The proportion of contaminated liquid cultures was determined over time.

For each Genotype MTBDR*plus* (HAIN Lifescience, Nehren, Germany) run performed between 2013 and 2017 the number of samples tested and the result of the negative control (positive or negative) were extracted from laboratory worksheets. The proportion of “positive”negative controls was calculated for each year and compared using χ^2^ test. Logistic regression was used to determine the odds ratios for PCR runs with evidence of DNA contamination (“positive” negative control) with 2013 serving as the baseline comparison.

All statistical analysis was performed using Stata version 14.2 (Texas, USA).

## Results

### Baseline reviews, actions and results

Table 1 summarizes the findings of the baseline reviews and the resulting actions across the twelve quality management essentials.

**Table 1:** Findings of the baseline reviews and the resulting action across twelve management essentials.

Quality system essential		Review findings	Actions	Outcomes
**(I)** **Facilities and safety**	Laboratory design, geographic and spatial organization	• Unrestricted access to BSL III • No sample reception (sample drop off in the hallway) • Crossing circulation pathways (biological samples, contamination waste, staff) • Lack of emergency exits Lack of delineation of laboratory activities • Suboptimal separation of pre-PCR, PCR and post-PCR processes	▪ Access limited to authorized staff ▪ Planning and securing finances for a new BSL III ▪ Optimising pathways within existing infrastructure ▪ Building of three additional emergency exits ▪ Clear delineation of laboratory activities through process control and infrastructural changes ▪ Separation of pre-PCR, PCR and post-PCR processes by providing additional bench space	Finance secured, BSL III design underway, with planned completion date Q3 2020 Decrease in DNA contamination from 38% to 8%
	Physical aspects of premises	• Inadequate physical infrastructure and inappropriate construction materials	▪ Planning and securing finances for a new BSL III	Finance secured, BSL III design underway, with planned completion date Q3 2020
	Safety management	• Rudimentary laboratory safety program • No designated safety officer • Limited standard safety practises and inadequate staff training • Unsafe waste management • Lack of autoclave validation and servicing • Ad hoc and unsupervised cleaning of the BSL III by locum cleaning staff • Inadequate fire safety equipment • Lack of gas alarm	▪ Development of a laboratory safety program ▪ Appointment of one of the technicians to become health & safety officer ▪ Implementation of standard safety practises in conjunction with extensive health and safety training ▪ Development of a waste management plan, procurement of appropriate equipment (i.e. autoclave boxes, transport vehicles) and consumables (waste containers) ▪ Autoclave validation for solid and liquid waste, establishment of a service contract ▪ Development of a cleaning plan and rota including laboratory staff only ▪ Upgrade of fire safety equipment ▪ Removal of bunsen burners and decommissioning of gas supply	Laboratory safety program and waste management plan established
	Identification of risk	• BSCs alterations potentially influencing airflow	▪ Refurbishment of BSCs	BSC refurbished
	Personal protective equipment	• No gloves policy	▪ Implementation of an “all gloves” policy ▪ Training to staff	
	Emergency management	• Lack of emergency management plan	▪ Development of an emergency management plan	
**(II)** **Equipment**	Troubleshooting, service, repair and retiring equipment	• Limited number of service contracts • Poor documentation of maintenance and service contracts	▪ Initiate service contracts ▪ Establish and maintain documentation	
	Equipment maintenance	• No equipment inventory • No equipment maintenance program • Limited BSC maintenance	▪ Compile equipment inventory ▪ Initiate an equipment maintenance program ▪ 6 monthly BSC maintenance	Equipment inventory completed
**(III)** **Purchasing and inventory**	Purchasing	• No purchasing process • Documentation of purchasing incomplete and variable	▪ Establish clear processes for selection, purchasing and receipt of supplies ▪ Introduce forms and logs to document purchasing and receipt of supplies	
	Inventory management	• No inventory management program	▪ Conduct an inventory serving as baseline and implement regular stock checks	Baseline inventory performed
	Storage of supplies	• Inadequate and non-standardised storage procedures	▪ Improve storage of consumable (i.e. regular temperatures checks)	
**(IV-1)** **Process control—sample management**	Laboratory handbook	• Available, but out-dated (i.e. new diagnostic methods missing)	▪ Update laboratory handbook	Updated laboratory handbook
	Sample processing	• Lack of sample rejection criteria, leading to all samples being processed regardless of suitability	▪ Establish procedures to assess quality of samples, introduce rules for rejecting samples and standardise feedback to referring clinicians	Sample rejection optimised
	Sample storage, retention and disposal	• Paper-based archiving system without temperature control (at -20C) • Storage of >20,000 cultures for up to 5 years in inadequate secondary storage containers with unrestricted access	▪ Computerised and temperature controlled (-80) archiving of cultured isolates in planning ▪ Deactivation of all cultures by autoclaving and safe disposal	Deactivation of old cultures completed
**(IV-2)** **Process control—quality control and method validation**	Quality control	• Quality control of stains, solid and liquid media (prepared in house) and drug stock solutions (prepared in house) not performed • Quality control of some commercially sourced media and drug stocks, but lack of standardised documentation	▪ Implementation of lot control for staining solutions, replacement of in house media by commercially sourced media ▪ Implementation of lot control documentation for all media	Verifications completed for NTM DST, fluorescence microscopy, Xpert^®^ MTB/RIF Ultra (Cepheid), FluoroType^®^ MTBDR (HAIN Lifescience), DST for new drugs (bedaquiline, clofazimine, delamanid)
	Method verification and validation	• Verification and validation not routinely performed	▪ Verification and validation of all newly introduced methods	
**(V)** **Assessment -audits**	External and internal audit	• External audits not performed • Internal audits not performed	▪ Not yet performed ▪ Initiation of an internal audit program starting with baseline reviews and some selected horizontal audits (such as temperature log keeping)	Baseline internal audit performed
	Proficiency testing	• Participation in the National German EQA, run by the NRL itself • Participation in the WHO EQA for first- and second-line DST for TB	▪ Participation in the US CAP (college of American pathology) EQA for microscopy, NAT, culture, identification and phenotypic DST	Successful participation of the first round of CAP EQA
	Certification and accreditation	• Laboratory not accredited	▪ Accreditation to ISO 15189 standard planned for 2020 (following the move into the new BSL III)	
**(VI)** **Personnel**	Recruitment and orientation	• No standardised process	▪ Development of SOPs for recruitment and orientation	
	Competency and competency assessment	• Competencies not recorded • Competency assessment not conducted	▪ Development of a competency matrix and processes on how to assess competence	Competency assessment reviewed and approved by workers’ council
	Training and continuing education	• No regular in-house training conducted • Limited external training opportunities for scientific staff members only	▪ Initiation of fortnightly internal training sessions covering pre-analytic requirements, analytic processes, documentation, information management, data protection, health & safety, fire safety ▪ Invitation of diagnostic companies to provide on-site training of new and established diagnostic methods ▪ Active encouragement and provision of funding for visits to other laboratories and external training for all staff members including technicians and administrators	27 internal group training sessions; Completion of three one to one training sessions for each staff member before implementation of the new LIS; 48 external training days; 25 visits to other laboratories; Completion of bachelor in laboratory management (acting laboratory manager); Completion of bachelor economic engineering (quality management technician); Completion of TB expert course (ECDC) (biomedical scientist)
	Employee performance appraisal	• Not performed	▪ Regular (yearly) employee appraisals by the clinical director	All staff have had two rounds of appraisals
**(VII)** **Customer Service**	Assessing and monitoring customer satisfaction	• No document of customers’ complaints or compliments • No processes for handling complaints	▪ Documentation of customers’ complaints and compliments	
**(VIII)** **Occurrence management**	Investigation of occurrences	• No process for investigating occurrences	▪ Implementation of a structured document to investigate occurrence ▪ Regular review of all occurrences by the quality manager and clinical director, followed by feedback and discussion with technical staff	25 occurrences logged since 16.6.2016 date, 23 occurrences closed, 2 open
	Rectifying and managing occurrences	• Ad hoc corrective actions without root cause analysis or lessons learned	▪ Staff training on how to instigate CAPA (corrective action and preventive action)	
**(IX)** **Process improvement**	Quality indicators	• Quality indicators not established	▪ Identification of a selected set of quality indicators ▪ Routine monthly data collection (initially extraction from paper records, later on as SQL queries)	Decrease in liquid culture contamination rates from 13–33% to <10% (Fig 2)
	Implementing process improvement	• No formal process	▪ Continuous process improvement through QM team leadership ▪ Regular feedback on QI during staff meetings and engagement of all lab staff	Decrease in liquid culture contamination rates from 13–33% to <10% (Fig 2)
**(X)** **Documents and records**	The quality manual	• No quality manual	▪ Development of a quality manual	
	Standard operating procedures (SOPs)	• No written SOPs	▪ Drafting of a SOP prototype ▪ Writing of SOPs in thematic blocks (i.e. microscopy, mycobacterial culture of primary samples)	Increase in smear positivity (Fig 1)
	Document control	• No system for document control	▪ Training on document control for NRL staff and other managerial staff in the research organization such as members of the workers’ council ▪ Implementation of a document control system	Document control system established
	Storing documents and records	• Storage of paper-based patient reports for 10 years	▪ Safe electronic storage of electronic records with daily back-up to the central server	
**(XI)** **Information management**	Computerized laboratory information systems (LIS)	• MS Access based LIS without functions of validation and verification, not adhering to national regulations	▪ Implementation of a LIS in line with statutory regulations ▪ Entering of referral forms using optical character recognition software and order entry systems	New LIS implemented and fully functioning with BSL III moving to near paperless laboratory
	Audit trails	• No audit trail of data entry available	▪ Data entry audit trail implemented as part of the LIS system	Audit trail of data entry fully implemented
	Invoicing	• Labour intensive manual invoicing without internal checks	▪ Invoicing through LIS with internal controls	Increase in revenue
	Data protection	• Institutional policy in place • No policy within the laboratory on whom to supply patient results to and how	▪ Training of all staff on data protection ▪ Policy on provision of laboratory results to clinician’s other than the referring clinician	Improved data protection in line with national regulations
**(XII)** **Organization**	Organization management and structure	• Autocratic leadership within a hierarchical structure • No clear lines of reporting • Roles and responsibilities not established	▪ Appointment of a new clinical director ▪ Promotion of one of the technicians to acting laboratory manager ▪ Organogram with clear lines of reporting ▪ Establishment of delineated roles and responsibilities with clear job descriptions and competency requirements	Sufficient, competent staff with appropriate authority and managerial oversight

Abbreviations: BSC–biosafety cabinet; BSL III–Biosafety laboratory III; CAP–College of American Pathologists; CAPA–corrective action and preventive action; DNA–Desoxyribonuleic acid; DST–drug susceptibility testing; ECDC—European Center for Disease Prevention and Control; EQA–external quality assessment; LIS–laboratory information system; NAT–Nucleic acid Amplification Test; NTM–non-tuberculous mycobacteria; NRL–National Reference Laboratory; PCR–Polymerase Chain Reaction; QI–quality improvement; QM–quality management; SOP–standard operating procedure; SQL–structured query language; TB–tuberculosis; WHO–World Health Organisation

Review of facilities and safety resulted in several infrastructural changes of the existing building including additional emergency exits, smoke detectors and fire alarms. Some infrastructural deficiencies were addressed by optimising and standardising processes such as sample flow and delineating laboratory activities. To improve safety a waste management plan was introduced and appropriate equipment (autoclave boxes) and consumables were purchased, a detailed cleaning plan was developed and staff were required to wear gloves in the BSL III. The feasibility study revealed that refurbishment of the existing building would not suffice to comply with the 2013 biosafety regulations. As a result, senior management sourced and secured financing for a new BSL III. Building permission was granted in September 2018 and completion is planned for 2020.

Documentation of equipment, purchasing and inventory was limited. Processes and documentation were agreed and an equipment and baseline stock inventory completed. Lack of sample rejection criteria meant that all samples were processed regardless of suitability. Minimal requirements for quality of samples were established, users informed and scientists and technicians trained. Standardised operating procedures and documentation for validation and verification of new methods were introduced. In total five new CE-marked methods (CE: *Conformité Européenne* [European conformity] with health, safety, and environmental protection standards for products sold within the European Economic Area) have been verified up to December 2017, and one method (DST for bedaquiline, delamanid and clofazimine) has been validated. Up to 2017 the NRL participated in the national EQA scheme led by the NRL itself. From 2018 the NRL took successfully part in the United States College of American Pathologists (US CAP) EQA for microscopy, NAT, culture, identification and phenotypic DST.

The majority of staff had been trained at the NRL and never worked at or visited other laboratories. The concept of quality management was unfamiliar to technical, scientific and administrative staff. Staff were encouraged to participate in training and visit other laboratories with the aim to create an environment of continuous learning, promote critical thinking and motivate for sustained change. A total of 25 laboratory visits by individual staff members took place over a period of two years. External training days totalled 48 (excluding the acting laboratory manager’s training days). In addition, diagnostic companies were invited to conduct on-the-job trainings in small groups. A one-hour internal fortnightly teaching session was implemented to introduce quality management concepts such as corrective and preventive actions, discuss SOPs under development and inform staff about future plans and changes. The implementation of the LIS was accompanied by intensive theoretical and practical training (three group sessions, and three individual training sessions for each staff member).

Components of the QMS were developed using a bottom-up approach wherever possible.

Quality indicators were not established and given the largely paper-based documentation of laboratory results were difficult to collect and analyse in real time. Initially a limited set of quality indicators (culture contamination rates, positive microscopy and NAT rates, discordances in microscopy, NAT and culture results) was identified to introduce the concept of quality indicator monitoring and continuous process improvement. Once the LIS was implemented in October 2017 trends of quality indicators were analysed on a monthly basis.

SOPs for analytical processes did not exist. Staff were using product inserts to perform diagnostics procedures. Because none of the technicians had ever drafted a SOP, the quality manager, clinical laboratory director and quality management technician did the actual writing of SOPs and validated the content with other staff members. The development of SOPs followed an iterative process: observation of the analytic process by the quality management team, drafting of the SOP, actively seeking feedback from the technicians, amendments and edits, multiple feedback rounds followed by the approval of the final SOP. Alongside the introduction of standardised processes and improved documentation a document control system was set up. All laboratory staff and the members of the workers council (mandatory elected body comprising elected employees with mandate to protect working conditions, health and safety and workers’ rights) were trained on document control. Laboratory documents were made available to the workers’ council on request to check compliance with working directives. Thus members of the workers’ council had to understand and adhere to document control procedures.

In mid 2015, an in-house access based (Microsoft 2013) LIS was introduced replacing a completely paper-based system, where results were entered on hard-copy sample sheets (one sheet per sample) and reports typed and signed. The access based LIS had several shortcomings and did not comply with national regulations. Features such as technical verification, medical authorization, audit trails, direct import of laboratory results from analysers, automated registration of samples via an optical character recognition (OCR) scanner and automated invoicing were not available. Technicians continued to use paper-based sample worksheets duplicating reporting. A new LIS (MLab, Dorner, Muehlhausen, Germany), OCR scanners for sample registration and automated result import from analysers was introduced in October 2017. Despite limited computer literacy of technical staff, the BSL III was able to move to near paperless result reporting. Registration of samples became less time consuming and error prone. Automated and standardised invoicing resulted in an increase in revenue and reduction of administrative staff time freeing 0.3 full time equivalents of administrator time.

### Smear microscopy

In 2015, smears were stained using a staining automate according to the manufactore´s instruction (Kreienbaum, Germany) and the Kinyoun staining method.[17] Briefly, the Kinyoun method refers to an acid-fast stain used to detect any species of the genus *Mycobacterium*. It involves the application of a primary stain (Carbol-Fuchsin), a decolorizer (acid-alcohol), and a counterstain (Armand Solution). In contrast to the classical Ziehl–Neelsen staining, the Kinyoun method does not require a heating step.

A review of the existing method revealed poor quality of smears and staining which included a high proportion of staining artefacts. As a result of the review it was decided to i) introduce fluorescence microscopy recommended by WHO [18] with a higher sensitivity [19] accompanied by changes such as the use of frosted slides and water filters to avoid contamination with environmental mycobacteria from tap water ii) remove the staining automate iii) develop SOPs iv) introduce and document batch controls of staining solution as well as positive and negative controls v) train technicians in smear preparation, staining and reading vi) assess competencies vii) routinely double-check any positive slide and viii) perform duplicate reading of ten randomly selected negative slides per week. These changes and the completion of all documents related to quality management of smear microscopy were implemented over a period of 3 months.

**Fig 1:**
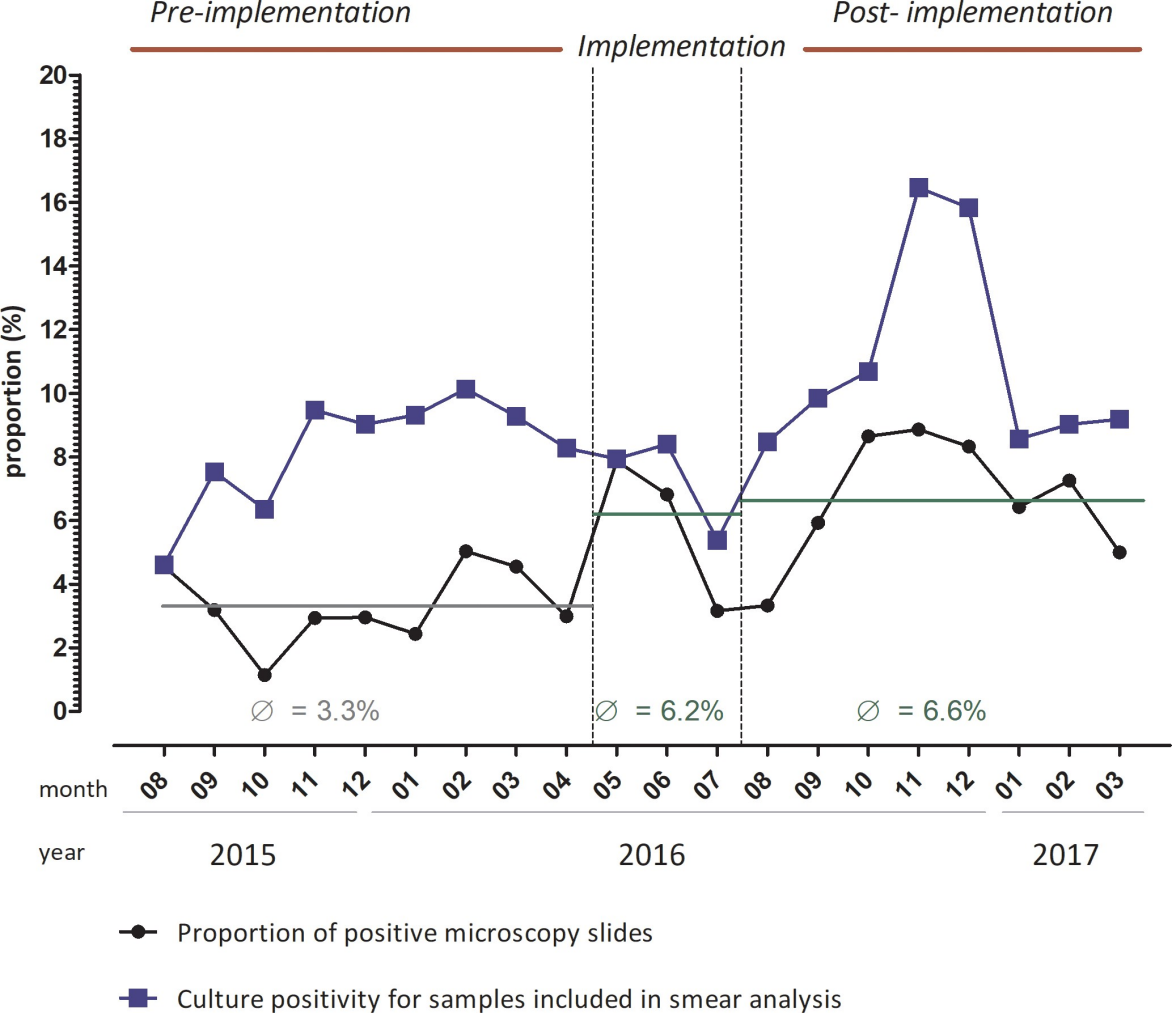
**Proportion of positive microscopy slides (black line) pre- implementation of quality control measures, during implementation and post-implementation (limited to the first respiratory sample of each patient). Culture positivity for samples included in smear analysis are shown (blue line).** Samples were comparable across different time periods. (DOI10.6084/m9.figshare.9882935) *Pre-implementation*: smears were stained using a staining automate and the Kinyoun staining method. *Implementation*: Fluorescence microscopy was introduced accompanied by using frosted slides and water filters. SOPs were developed including e.g. documentation of batch controls of staining solution, training of technicians and introduction of positive and negative smear controls. *Post-implementation*: Quality control measures were continued and technicians were re-trained at 6 monthly intervals.

Before implementation of quality managed fluorescence microscopy the proportion of respiratory samples classified as smear positive was 3.3% (Fig 1). During the implementation and post-implementation period the proportion of respiratory samples testing smear positive increased to 6.2% and 6.6% respectively (p-value <0.01). The odds ratio for a sample to be tested as microscopically positive increased by 1.94 (95% CI 1.16–3.26, p = 0.012) and 2.08 (95%CI 1.41–3.06, p <0.001) comparing the time before the interventions with the scale-up phase and the time when quality managed fluorescence microscopy was fully implemented. The proportion of false positives was 0.02%, 0.01%, 0.02% before, during and post-implementation of the interventions respectively.

### Culture contamination rates

**Fig 2:**
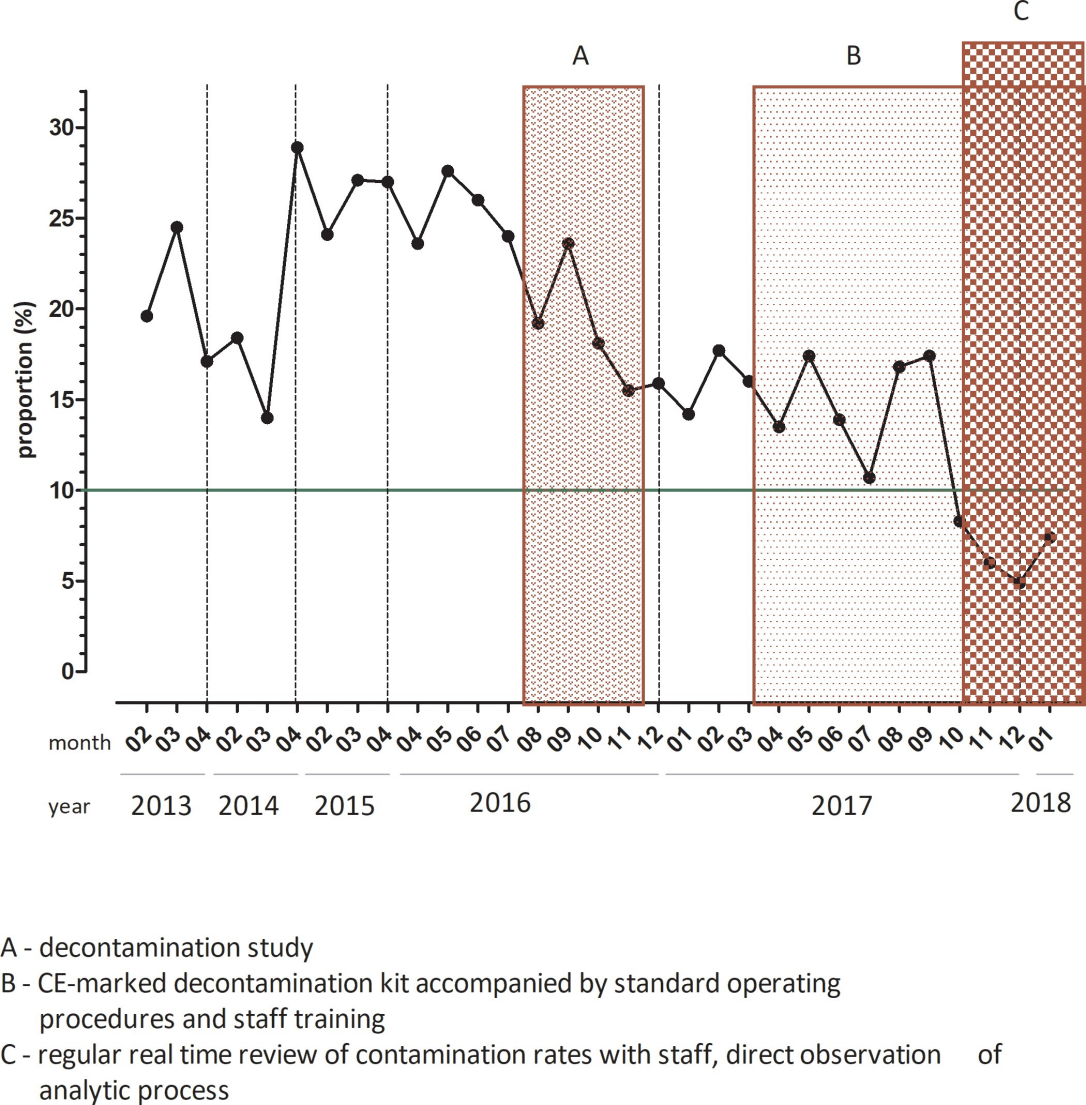
Contamination rates in liquid culture over time. Baseline reviews showed that contamination rates between the years 2013–2015 exceeded the targets of <10% in liquid media (3 representative months/year are shown). Between 2016 and 2017, different approaches (A–decontamination study; B–CE-marked decontamination kit accompanied by standard operating procedures and staff training, C–regular real time review of contamination rates with staff, direct observation of analytic process) were applied and impact on contamination rates were measured.

Baseline reviews showed that contamination rates in previous years (2013–2015) exceeded the target of <10% in liquid media set by the national German microbiology standards (Fig 2).[16] This resulted in the implementation of several measures aimed at improving quality. First the results of the retrospective assessment were presented and discussed at one of the forth-nightly laboratory meetings. Contamination rates were selected as one of the first quality indicators to be monitored and data were extracted manually from paper records from April 2016 onwards. However, because the process was not automated contamination rates were not available in real-time.

In August 2016, a study comparing different decontamination methods (three commercially available methods and the in-house method) was conducted over a period of 10 weeks.[20] For each method short SOPs were developed, technicians were trained and following competency assessments they were observed performing the decontamination procedures. This led to a temporary reduction of contamination rates among routinely processed samples during the study period (Fig 2). Finally, in March 2017 the decision was taken to implement a CE-marked commercially available method based on N-Acetyl-L-Cysteine-Sodium Hydroxide (NALC-NAOH, MycoDDRTM, IMMY, Norman, USA). Following the implementation of a new LIS in August-September 2017, quality indicators and statistics were generated automatically by Structured Query Language (SQL) queries. Weekly meetings were held to review contamination rates with all staff members involved in processing primary samples. Together with the technicians performing the decontamination procedures prompts and aids were developed to ensure correct incubation times and sufficient vortexing. Graphs plotting contamination rates over time were displayed in the staff room. Over a period of two weeks, decontamination procedures were directly observed on five separate days by the acting laboratory manager. Contamination rates decreased from 23.6–27.6% in April-July 2016 to 10.7–17.4% in the same period in 2017 and fell under the 10% mark in November 2017-March 2018.

### DNA contamination

In 2015, the Xpert MTB/Rif (Cepheid, USA) was the main molecular diagnostic used for detection of resistance referring mutations at the NRL. In contrast, the number of samples investigated using the Genotype MTBDR*plus* was limited, because DNA contamination was a frequently observed event. Strict separation of pre-PCR, PCR and post-PCR processes was difficult due to suboptimal building design and sample flow. Further challenges included non-directional airflow, inadequate staff training on how to prevent DNA contamination and a culture of austerity aimed at saving consumables and time. Over a period of several months, additional bench and storage space was built to allow separation of pre-PCR, PCR and post-PCR processes. In addition, room access with regards to whom (staff) and when (time of the day) and cleaning procedures were discussed with scientific and technical staff and agreed upon. Any PCR run with a “positive” negative control was brought to the attention of senior management and the run was repeated. If still positive on the repeat run, intensive cleaning was conducted, new reagents and pipettes were sourced.

Between 2013 and 2017 a total of 610 individual Genotype MDR*plus* diagnostic runs were performed investigating a total of 1780 samples. The median number of samples tested per run increased from one in 2013 and 2014 to two, three and five in 2015, 2016 and 2017 respectively (Table 2), while the proportion of negative controls showing evidence of DNA contamination decreased from 38.2% in 2013 to 8.1% in 2017 (p value <0.01). The odds ratio for a run with evidence of DNA contamination was 0.91 (95%CI 0.53–1.57) for 2014 compared to 2013, but decreased for the subsequent years to 0.42 (95%CI 0.23–0.75), 0.28 (95%CI 0.15–0.54) and 0.14 (95%CI 0.07–0.29).

**Table 2:** Summary of Genotype MDR*plus* tests performed between 2013 and 2017.

Year	Total number of runs	Median (IQR) number of diagnostic samples per run	Mean number of diagnostic samples per run	Proportion (95%CI) of negative controls showing evidence of contamination	Odds ratio (95%CI) for a negative control showing evidence of contamination
2013	116	1 (1; 1)	1.33	38.2% (29.1; 47.9)	1
2014	112	1 (1; 2)	1.61	36.0% (27.1; 45.7)	0.91 (0.53; 1.57)
2015	127	2 (1; 3)	2.15	20.6% (13.9; 28.8)	0.42 (0.23; 0.75)
2016	115	3 (2; 5)	3.33	14.9% (8.9; 22.8)	0.28 (0.15; 0.54)
2017	140	5 (3; 7.5)	5.64	8.1% (4.1; 14.0)	0.14 (0.07; 0.29)

CI: confidence interval; IQR interquartile range.

**Fig 3:**
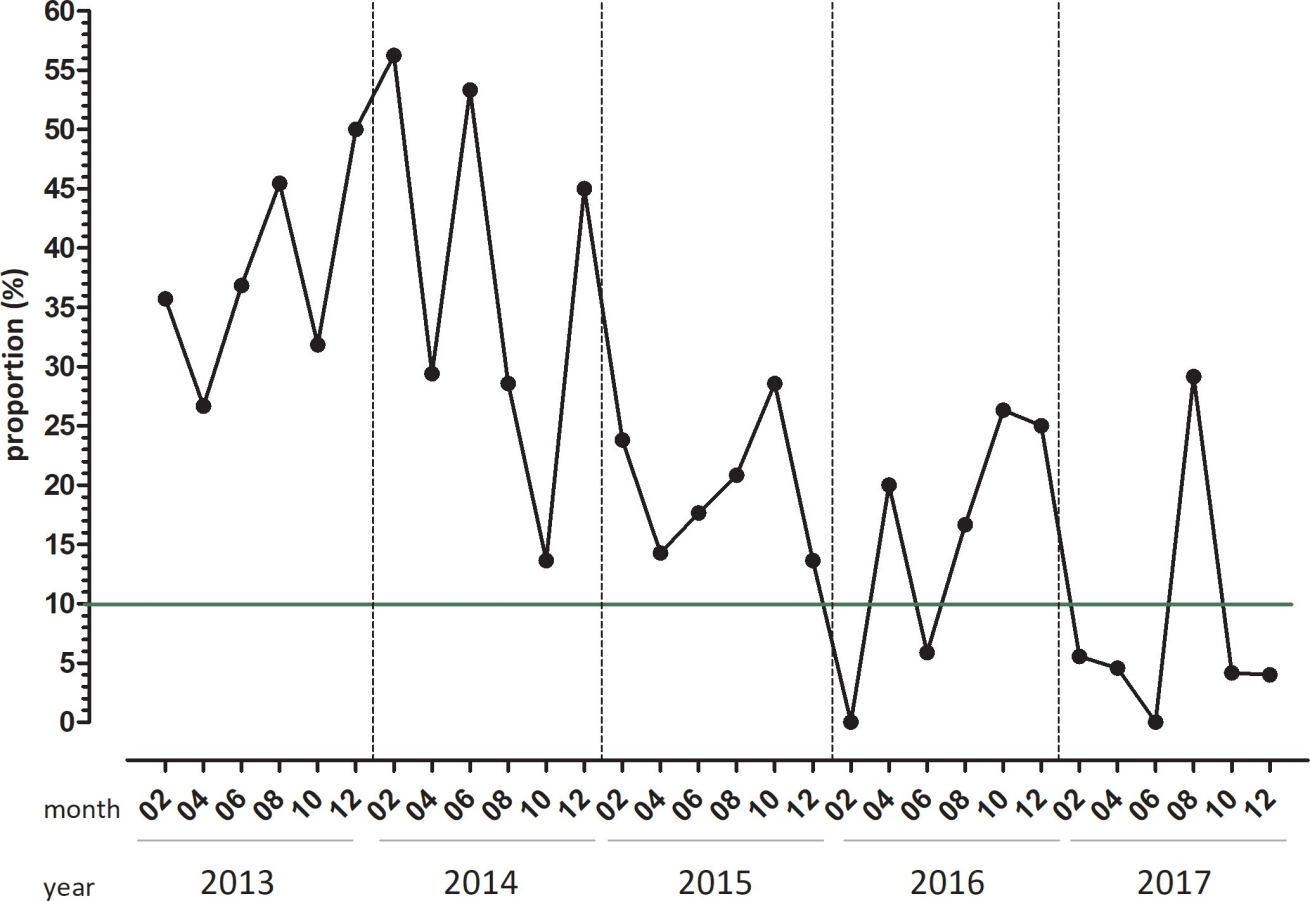
Proportion of PCR runs with evidence of DNA contamination over 5 years. Between 2013 and 2017 a total of 610 PCR runs were performed. The proportion of negative controls showing evidence of DNA contamination decreased from 38.2% in 2013 to 8.1% in 2017 by separating pre-PCR and post-PCR processes followed by staff training. (DOI 10.6084/m9.figshare.9882935).

Of the 11 runs with “positive” negative controls in 2017 seven occurred in July and August (Fig 3). These triggered corrective and preventive actions including repeat testing of the clinical specimens, replacement of pipettes and mastermix and intensive cleaning. These measures decreased DNA contamination immediately (4.1%; 95%CI 0.49–13.98). Excluding the months of July and August DNA contamination was evident in 3.6% (95%CI 0.98–8.89) runs in 2017.

## Discussion

This study demonstrates the impact of quality management in a National TB reference laboratory in a high-income country across different methods: smear microscopy, culture and molecular diagnostics. Implementation of quality managed fluorescence microscopy doubled the yield in respiratory samples, while contamination rates in liquid media decreased significantly from over 20% at the beginning of this study to less than 10% during the most recent follow-up. Retrospective data collection showed evidence of DNA contamination in more than a third of PCR runs in 2013 and 2014. The odds of DNA contamination decreased by 85% comparing 2013 data with 2017 data.

While QMS implementation has not been completed and the laboratory is not yet accredited, the results show significant improvements due to introduction of quality management processes. In parallel with the implementation of a QMS and LIS finances have been secured to build a new BSL III. The decision has been taken to defer accreditation until the new laboratory has been completed. This is currently planned for 2020. Publication of our experience and these findings at this time prior to reaching accreditation serves two purposes. First our data-driven approach by implementing measurement of the impact of QMS using trend analysis of quality indicators has already demonstrated substantial benefit. The analysis has been a key enabler to motivate for the additional investment needed. Secondly, although accreditation is our eventual aim, laboratories considering improvement of QMS can follow our approach with or without accreditation as the final goal.

Success in attaining sustained improvement cannot be attributed to a single measure. A combination of committed senior leadership, significant financial resources and a bottom-up approach was crucial for these achievements. In addition, a non-blame culture of continuous improvement has resulted in enhanced staff competency and fostered their ability of critical appraisal, improved operational consistency and reliability and teamwork. The baseline review included interviews with all staff members and revealed that most of them were afraid to make any mistakes and even more of being caught. A two-day team building exercise in February 2016 showed that lack of skill and understanding of analytic procedures impeded effective teamwork and communication. Therefore, a special emphasis was placed on training, exposure to other laboratories and communication through in-house seminars and daily staff meetings.

Unfortunately, no formal staff feedback was sought. However, a study conducted in three laboratories in the United Kingdom reporting that the majority of laboratory technicians thought the documentation required for accreditation increased the workload without improving quality of test results.[21] In contrast, laboratory managers and clinicians in the United Kingdom and South Korea felt that accreditation resulted in better laboratory performance with more documentation, better health and safety and training procedures and improved infrastructure.[22, 23]

There seems widespread agreement that laboratory quality management is important for patient safety and impacts on quality of clinical care.[4, 11] However, studies reporting on the impact of full-scale implementation of QMS and/or accreditation on laboratory performance are relatively few.[9, 10, 24–26] We have recently conducted a systematic review showing that quality improvement measures had indeed a measurable impact in TB laboratories in low- and lower-middle income countries.[27] Only one of the studies included in the review reported on the effect of implementing a comprehensive QMS followed by accreditation.[9] All other studies described the effect of various activities aimed at improving quality such as EQAs, supervisory visits and staff training.

One of the main issues of measuring the effect of QMS is the measurement of quality before implementing QMS. Data collection on quality indicators requires partial QMS implementation. This in turn makes it difficult to assess the full impact of QMS on quality indicators.

In this study, pre-intervention data were collected retrospectively. Hence data collection of quality indicators itself could not have impacted on performance. Outcomes in this study were analyzed using an uncontrolled before-and-after comparison, which is always vulnerable to coincidental time trends. However, impact was measured across three different analytic tests over a prolonged time period. Thus changes due to seasonal variations or differences in patient population are unlikely to explain the significant differences detected over time.

Unfortunately, we did not collect any data on cost and hence were unable to calculate cost-effectiveness or report on any cost-savings.

Over the past decade the need to improve laboratory quality in low and middle-income countries has been widely acknowledged.[28–30] The Strengthening Laboratory Management Toward Accreditation (SLMTA) initiative launched in 2009 has had a substantive impact on provision of quality laboratory services and patient care.[31–34] However, this study shows that quality management is effective regardless of low or high resource settings. It serves as a stark reminder that while quality management is worth our while it is cost and resource intensive and requires full commitment from senior management.
